# 非小细胞肺癌患者术后发生骨转移的危险因素

**DOI:** 10.3779/j.issn.1009-3419.2018.06.09

**Published:** 2018-06-20

**Authors:** 源山 姚, 银杰 周, 振华 杨, 海波 沈

**Affiliations:** 315010 宁波，宁波市第二医院胸外科 Department of Thoracic Surgery, Ningbo No.2 Hospital, Ningbo 315010, China

**Keywords:** 肺肿瘤, 骨转移, 危险因素, Lung neoplasms, Bone metastasis, Risk factors

## Abstract

**背景与目的:**

肺癌目前是死亡率最高的肿瘤, 非小细胞肺癌(non-small cell lung cancer, NSCLC)患者在手术后通常会发生远处转移, 如骨转移、脑转移、肺转移等。本研究旨在探究NSCLC患者术后发生骨转移的危险因素。

**方法:**

选择本院于2009年5月-2011年5月确诊收治的NSCLC患者176例, 按照是否发生骨转移将患者分为两组, 即骨转移组和无骨转移组。对比两组患者的一般临床病理资料, 并通过多因素分析对比发生骨转移的独立危险因素。

**结果:**

NSCLC患者的一般临床病理资料中血栓与否, 肿瘤-淋巴结-转移(tumor-node-metastasis, TNM)分期与是否发生骨转移关系密切, 有统计学意义(均*P* < 0.01);在两组患者的凝血功能指标中发现凝血酶原时间、活化部分凝血酶活酶时间、纤维蛋白原、凝血酶时间、血小板计数、D-二聚体以及碱性磷酸酶之间存在明显的差异性, 有统计学意义(均*P* < 0.05);*Logistic*回归分析发现纤维蛋白原、碱性磷酸酶、T4期、N3期和D-二聚体为NSCLC患者发生骨转移的独立危险因素。

**结论:**

纤维蛋白原、活化部分凝血酶活酶时间、碱性磷酸酶、T3期、N2期和D-二聚体为NSCLC患者发生骨转移的独立危险因素。

## Abstract (en)

**Background and objective:**

Lung cancer is the leading cause of cancer-related deaths, patients with non-small cell lung cancer (NSCLC) usually have distant metastases, such as bone metastasis, brain metastasis, and lung metastasis.The purpose of this study was to explore the risk factors for bone metastasis in NSCLC patients.

**Methods:**

A total of 176 cases of NSCLC were selected from May 2009 to May 2011, and patients were divided into two groups, namely the bone metastasis group and non-bone metastasis group.The general clinicopathological data of the two groups and analyzing the independent risk factors of bone metastasis were compared.

**Results:**

In the general clinicopathological data of NSCLC patients.The thrombus or not and tumor-node-metastasis (TNM) stage were closely related to the occurrence of bone metastasis, and were statistically significant (all *P* < 0.01).Prothrombin time, activated partial thromboplastin time, Fibrinogen, thrombin time, blood platelet, D-Dimer and alkaline phosphatase have significantly difference between the two groups (all *P* < 0.05).*Logistic* regression analysis showed that fibrinogen, activated partial thromboplast in time, alkaline phosphatase, T4 phase, N3 phase and d-dimer were independent risk factors for bone metastasis in NSCLC patients.

**Conclusion:**

Fibrinogen, alkaline phosphatase, T3, N2 stage and D-Dimer is the independent risk factors of bone metastases in patients with NSCLC.

肺癌目前在全球的发病率高居首位, 其死亡率也最高^[1]^。全球每年死于肺癌的患者接近200万。环境污染的增加, 肺癌的发病人数逐年递增, 单就亚洲每年新增的肺癌患者已超过50%^[2]^, 给患者及家庭带来巨大的负担和痛苦。非小细胞肺癌(non-small cell lung cancer, NSCLC)患者人数占比超过所有肺癌患者人数的85%, 而这其中又以腺癌和鳞状细胞癌最为常见, 占比超过肺癌患者人数的65%^[3, 4]^。肺癌患者在早期时没有显著的体征表现, 一旦确诊为肺癌时往往处于晚期, 因此治愈的难度极大。癌症患者的死亡通常是由于复发或者转移引起的, 骨组织是机体内最为主要的血行转移部位之一^[5]^, 是除了肺和肝脏之外最常见转移位置, 骨转移常累及中轴骨骼等部位^[6]^。临床上经常使用全身骨扫描对骨转移进行诊断, 但费用高, 技术水平要求高, 所以医院的普及率还比较低。目前还没有独立有效的可以检测骨转移发生的血清生化指标^[7, 8]^。本研究旨在对NSCLC患者在术后发生骨转移的危险因素进行分析, 并随访评估患者的预后, 以期望能够为临床治疗提供参考。

## 1 资料与方法

### 1.1 一般资料

选择本院2009年5月-2011年5月确诊收治的NSCLC患者176例, 按照是否发生骨转移将患者分为两组, 即骨转移组和非骨转移组。

纳入标准:组织病理学明确诊断为肺腺癌或鳞状细胞癌的患者; 在本院确诊前半年内没有经历过抗肿瘤治疗史。骨转移患者经过正电子发射型计算机断层显像(positron emission computed tomography, PET)-计算机断层扫描(computed tomography, CT)/磁共振成像(magnetic resonance imaging, MRI)证实为骨转移, 非骨转移患者经胸部及上腹部CT、头颅MRI、PET-CT及B超等检查明确肺、肝、肾上腺、脑、淋巴结及皮下等部位无转移, 下肢静脉超声判断患者是否发生血栓。

排除标准:以往存在肝肾疾病或出现肝肾上腺转移的患者; 存在对骨代谢有影响的内分泌系统疾病; 存在血小板减少性紫癜等血液系统疾病; 在1年内发生骨折等骨骼良性病变的患者。

### 1.2 方法

对比分析NSCLC患者的一般临床病理资料和凝血功能指标, 如凝血酶原时间(prothrombin time, PT)、凝血酶原活动度(prothrombin time activity, PTA)、国际标准化比值(international normalized ratio, IRN)、纤维蛋白原(fibrinogen, FIB)、活化部分凝血活酶时间(activated partial thromboplastin time, APTT)、凝血酶时间(thrombin time, TT)、部分凝血酶比率(partial thrombin ratio, PTR)、凝血酶比率(prothrombin ratio, PR)、血小板计数(blood platelet, PLT)、碱性磷酸酶(alkaline phosphatase, AKP)和D-二聚体(D-Dimer)。确认各类资料与骨转移的相关性, 并通过多因素分析骨转移患者的独立危险因素。

### 1.3 统计学分析

采用SPSS 20.0软件进行统计学分析, 计数资料采用率(%)表示, 组间比较采用χ^2^检验; 计量资料采用均数±标准差(Mean±SD)表示, 组间比较采用*t*检验, 采用*Logistic*回归法分析骨转移的独立危险因素, 以α=0.05为检验统计标准。

## 2 结果

### 2.1 两组患者一般临床病理特征对比

性别、年龄、病理类型、是否吸烟及手术方式与患者发生骨转移之间没有必然的关联性, 无统计学意义(均*P* > 0.05), 而是否发生血栓, 肿瘤-淋巴结-转移(tumor-node-metastasis, TNM)分期等因素与骨转移之间关系非常密切, 存在明显的统计学意义(均*P* < 0.01)。

### 2.2 两组患者凝血功能指标对比

两组患者凝血功能指标对比分析发现PT、FIB、TT、PLT、D-Dimer以及AKP之间存在明显的差异性, 有统计学意义(均*P* < 0.05)。

### 2.3 *Logistic*回归分析

将骨转移情况作为因变量Y, 发生则定义Y=1, 未发生则定义Y=0。将χ^2^检验和*t*检验中出现差异性表达的指标作为自变量X, 并将其赋值。发生血栓、T4期、N2期-N3期、APTT高于42 s, FIB高于4.4 g/L、PLT高于300×10^9^/L、女性AKP高于135 U/L、男性AKP高于125 U/L、D-二聚体高于0.23 mg/L均赋值为1, 其他相对于的分组均赋值为0。

结果显示*Logistic*回归方程的独立危险因素总有六项, 即FIB、APTT, AKP、T4期、N2期-N3期和D-二聚体, 见表 3。

**3:** 多因素分析对比 Comparative analysis of multiple factors

	B	Wald	*P*	Exp (B)	OR (95%CI)
Thrombus	1.794	2.885	0.082	6.147	0.785-50.482
FIB	0.913	7.187	0.003	2.685	1.295-5.861
APTT	-1.196	4.729	0.028	0.298	0.106-0.942
AKP	1.385	13.957	0.000	4.184	1.957-8.618
T4	1.031	7.738	0.006	4.152	1.382-5.295
N2-N3	0.804	3.749	0.048	2.524	1.037-5.023
D-Dimer	2.795	33.041	0.000	12.047	5.203-28.461
Constant term	-2.644	22.385	0.000		

## 3 讨论

目前我国已经成为全球恶性肿瘤新增病例最多的国家之一, 我国2015年肺癌的发病人数和死亡人数分别为82.1万例, 64.8万例^[9]^。目前普遍认为我国的肺癌患者与空气污染、吸烟、接触致癌物等因素有关联, 其中NSCLC是最为常见的肺癌, 其中又以腺癌和鳞状细胞癌最为常见。随着医疗技术的不断发展, 肺癌患者的生存期得以延长, 现在越来越多的集中在于肺癌相关联的并发症, 又以骨转移最为突出^[10, 11]^。

**1:** 两组患者一般临床病理特征对比 Comparison of general clinicopathologic features between the two groups

	Bone metastasis group (*n*=37)	Non-bone metastasis group (*n*=139)	χ^2^	*P*
Sex			0.001	0.968
Male	26	95		
Female	11	44		
Age (yr)			3.305	0.072
≤65	30	98		
> 65	7	41		
Pathology			3.175	0.087
Squamous carcinoma	8	42		
Adenocarcinoma	29	97		
Smoking			0.104	0.761
Yes	19	68		
No	18	71		
Surgical procedures			0.163	0.681
Thoracotomy	20	55		
Thoracoscope	17	84		
Thrombus			11.664	0.001
Yes	3	1		
No	34	138		
T stage			14.027	0.000
T1-T3 stage	25	44		
T4 stage	12	95		
N stage			19.385	0.000
N0-N1 stage	29	50		
N2-N3 stage	8	89		

**2:** 两组患者凝血功能指标对比(Mean±SD) Comparison of coagulation function between the two groups (Mean±SD)

	Bone metastasis group (*n*=37)	Non-bone metastasis group (*n*=139)	*t*	*P*
PT	10.03±1.22	11.35±1.74	-4.613	0.000
PTA	92.94±18.47	100.31±45.26	-1.037	0.317
INR	1.07±0.11	1.09±0.14	0.015	0.896
FIB	4.55±0.92	3.96±1.03	2.016	0.041
TT	13.06±1.48	14.22±1.95	-2.547	0.013
PTR	1.03±0.24	0.95±0.19	1.089	0.292
PR	0.94±0.11	0.95±0.13	-0.649	0.457
PLT	290.47±112.47	258.43±95.02	1.957	0.044
AKP	109.46±52.41	94.06±41.33	3.264	0.001
D-Dimer	0.54±0.42	0.18±0.11	12.056	0.000

有资料^[12]^表明目前有超过30%的NSCLC患者发生骨转移。近年来许多学者发现在很多肿瘤患者的凝血功能发生异常, 且部分表现出高凝, 容易形成血栓, 这是肿瘤细胞能够进行血行转移的先决条件^[13, 14]^。本研究结果显示血栓与否在骨转移和非骨转移患者中有较为显著的差异性(χ^2^=11.664, *P*=0.001), 这提示当肿瘤患者体内发生血栓时, 更易发生骨转移。Ayan等^[15]^认为T3期时NSCLC患者发生骨转移的独立因子之一, N2分期与较高的转移风险有显著的正相关性。本研究结果发现当处于T1-T3和N0-N1期时, 骨转移组和非转移组对比差异明显, 说明在此阶段患者更容易发生骨转移。发生骨转移时会引起骨骼受损, 成骨细胞会分泌碱性磷酸酶, 以修复受损的骨骼^[16, 17]^。由于很多因素可导致其活性升高, 故而在本研究中首先排除这类因素, 确保纳入标准。本研究结果发现碱性磷酸酶活性在NSCLC骨转移患者血清中的表达明显升高(*t*=3.264, *P*=0.001), 这与多数研究结果相吻合^[18, 19]^。

已经发现纤维蛋白原在凝血酶作用下变成纤维蛋白单体, 然后进一步交联成纤维蛋白。研究发现当纤维蛋白含量升高至一定水平时, 将会对血管内皮细胞产生不可逆的损伤作用, 同时还会促进血液中血小板的聚集。纤维蛋白原是一种急性期的应激蛋白, Riihimäki等^[20]^发现纤维蛋白原与恶性实体肿瘤的转移之间存在密切的关联性, 主要是起到桥梁的作用, 能够使得白细胞含量上升, 血小板和肿瘤细胞间的粘附结合, 可以保护肿瘤细胞免受损伤。活化部分凝血酶活酶时间主要反映机体内源性凝血功能是否正常, 当时间缩短时, 提示机体处于高凝血状态, 可能机体正处于血栓性疾病。D-二聚体则反映了机体内纤维蛋白的溶解功能, D-二聚体数值升高则表明机体呈高凝状态、继发性纤溶亢进、弥散性血管内凝血等疾病。本研究中通过检测患者的各项凝血功能指标发现凝血酶原时间、活化部分凝血酶活酶时间、纤维蛋白原、凝血酶时间、血小板计数、D-二聚体在NSCLC患者骨转移组和非骨转移组中差异较为明显, 有统计学意义(均*P* < 0.05)。因此我们推测当NSCLC患者处于高凝状态时更加容易发生骨转移现象。将与骨转移有关联的各项指标纳入*Logistic*回归方程, 分析发现NSCLC患者术后发生骨转移的独立危险因素有纤维蛋白原、活化部分凝血酶活酶时间、碱性磷酸酶、T4期、N2-N3期和D-二聚体。然而本研究还存在着一些不足, 如数据偏倚及不可避免的误差, 样本量对结果的客观性造成影响, 样本量越大则各期的病例越均衡, 研究的结果越全面可靠, 这些在后期的研究中要进一步加强。

综上所述, 纤维蛋白原、活化部分凝血酶活酶时间、碱性磷酸酶、T4期、N2期-N3期和D-二聚体为NSCLC患者发生骨转移的独立危险因素, 应当引起临床医生的足够重视。
